# Diacerein Improves Left Ventricular Remodeling and Cardiac Function by Reducing the Inflammatory Response after Myocardial Infarction

**DOI:** 10.1371/journal.pone.0121842

**Published:** 2015-03-27

**Authors:** Anali Galluce Torina, Karla Reichert, Fany Lima, Karlos Alexandre de Souza Vilarinho, Pedro Paulo Martins de Oliveira, Helison Rafael Pereira do Carmo, Daniela Diógenes de Carvalho, Mário José Abdalla Saad, Andrei Carvalho Sposito, Orlando Petrucci

**Affiliations:** 1 Laboratory of Myocardial Ischemia/Reperfusion, Faculty of Medical Science, State University of Campinas—UNICAMP, Campinas, SP, Brazil; 2 Department of Surgery, Discipline of Cardiac Surgery, Faculty of Medical Science, State University of Campinas—UNICAMP, Campinas, SP, Brazil; 3 Department of Internal Medicine, Faculty of Medical Science, State University of Campinas—UNICAMP, Campinas, SP, Brazil; National Institutes of Health, UNITED STATES

## Abstract

**Background:**

The inflammatory response has been implicated in the pathogenesis of left ventricular (LV) remodeling after myocardial infarction (MI). An anthraquinone compound with anti-inflammatory properties, diacerein inhibits the synthesis and activity of pro-inflammatory cytokines, such as tumor necrosis factor and interleukins 1 and 6. The purpose of this study was to investigate the effects of diacerein on ventricular remodeling *in vivo*.

**Methods and Results:**

Ligation of the left anterior descending artery was used to induce MI in an experimental rat model. Rats were divided into two groups: a control group that received saline solution (n = 16) and a group that received diacerein (80 mg/kg) daily (n = 10). After 4 weeks, the LV volume, cellular signaling, caspase 3 activity, and nuclear factor kappa B (NF-κB) transcription were compared between the two groups. After 4 weeks, end-diastolic and end-systolic LV volumes were reduced in the treatment group compared to the control group (p < .01 and p < .01, respectively). Compared to control rats, diacerein-treated rats exhibited less fibrosis in the LV (14.65%± 7.27% vs. 22.57%± 8.94%; p < .01), lower levels of caspase-3 activity, and lower levels of NF-κB p65 transcription.

**Conclusions:**

Treatment with diacerein once a day for 4 weeks after MI improved ventricular remodeling by promoting lower end-systolic and end-diastolic LV volumes. Diacerein also reduced fibrosis in the LV. These effects might be associated with partial blockage of the NF-κB pathway.

## Introduction

Myocardial infarction (MI) is a devastating event, especially when reperfusion does not occur[1, 2]. Left ventricular (LV) remodeling after MI involves enlargement of the LV and thinning of the ventricular wall to maintain cardiac function[3]. The inflammatory response plays an important role in LV remodeling [4]. For example, reperfusion injury can trigger a cascade of signaling events that lead to inflammatory tissue damage. These signaling factors can include pro-inflammatory cytokines, such as tumor-necrosis factor α (TNF-α), anti-inflammatory molecules, adhesion molecules, and interleukins[5, 6].

TNF-α is a pro-inflammatory cytokine that participates in the innate immune system and is expressed by cardiac tissues. At low levels, TNF-α exhibits a cardioprotective effect, whereas at high levels, TNF-α has been shown to mediate detrimental effects [6, 7]. TNF-α has been implicated in the pathogenesis of ventricular remodeling after MI [5, 8]. In clinical trials, blocking or decreasing the bioavailability of TNF-α produced disappointing results in patients with congestive heart failure [8, 9]. However, these patients had already undergone remodeling and dilation of the LV, and the results may have been affected by the dichotomous effects of TNF-α.

To our knowledge, no clinical trial to date has evaluated the effects of anti-inflammatory intervention on LV remodeling immediately after MI. However, there is some evidence that inhibition of nuclear factor kappa B F-κB) improves LV remodeling and contributes to a decrease in cardiac dysfunction after MI. Moreover, NF-κB is regulated, in part, by TNF-α [8, 10, 11].

Diacerein is an anthraquinone compound with anti-inflammatory properties that inhibit the synthesis and activity of pro-inflammatory cytokines, such as TNF-α and interleukins 1 and 6 (IL-1 and IL-6, respectively)[12–14]. The active metabolite for diacereinis rhein (1,8-dihydroxy-3-carboxyanthraquinone), which is found in plants of the genus *Cassia* and has exhibited anti-inflammatory effects by inhibiting cytokine synthesis [14]. In the present study, LV remodeling was evaluated in the presence ofdiacerein4 weeks after MI was induced in a rat model.

## Methods

### Animals and Ethics Statement

Wistar rats had free access to water and a standard rat diet (State University of Campinas Central Breeding Center, Campinas, Brazil), and were housed in a room maintained at 21°C with a 12-hour light/12-hourdark cycle. All experimental protocols were established in accordance with the standards of the Brazilian Council in Animal Experimentation, and the “Guide for Care and Use of Laboratory Animals” published by the US National Institutes of Health (NIH Publication No. 85–23, revised 1996). The protocol was approved by the Institutional Committee on the Ethics of Animal Experiments of the State University of Campinas under the permit number 2428–1.

### Induction of MI and Study Protocol

Male Wistar rats (120–150g) were subjected to an MI event according to the method of Gao et al.[15]. Briefly, rats were anesthetized with inhalation of 2% isoflurane with no endotracheal tube placement. A thoracic incision was made over the left region of the chest, and a purse string suture was made in the skin for incision closure at the end of the procedure. The thorax was accessed through the fourth intercostal space, and the heart was gently popped out through the incision.

MI was induced by performing a left coronary artery (LCA) ligation approximately 3 mm from its origin using a 6–0 polypropylene suture. After ligation, the heart was immediately placed back into the chest, manual evacuation of air was performed, and the suture was closed by snaring the previously placed purse string suture in the skin. If necessary, a needle was inserted into the eighth intercostal space to remove any residual pneumothorax. Rats were subsequently provided 40% oxygen and were monitored during recovery. All animals received acetaminophen 200 mg.kg^-1^ per os for 3 days.

After induction of MI and total recovery from anesthesia, animals were treated daily for 4 weeks with either 80 mg/kg diacerein diluted with 2 ml of saline solution (Diacerein group; n = 10) or a gavage of 2 ml of saline solution every day (Control group; n = 16). The diacerein dose applied was selected based on previous studies [14, 16, 17]and unpublished data from our laboratory. Two Sham groups were performed in the same way as described above with exception of no MI was induced by LCA ligation. One group was treated daily for 4 weeks with a gavage of 2 ml of saline solution (Sham Group; n = 8) and the second group was treated daily for 4 weeks with 80mg/kg diacerein diluted with 2 ml of saline solution (Sham group with Diacerein; n = 10). Body weights were recorded each week, and the diacerein dose administered was corrected for changes in weight. After 4 weeks, animals were submitted to a hemodynamic study and euthanasia for tissue harvesting. The euthanasia was induced with pentobarbital 100 mg.kg^-1^ and confirmed by the LV catheter inserted during the hemodynamic assessment.

### Hemodynamic Assessment

Four weeks after the MI procedure, rats were anesthetized with xylazine (5 mg/kg) and ketamine (75 mg/kg) by intraperitoneal injection and were allowed to breathe spontaneously. A hemodynamic invasive assay was performed by using apressure-volume catheter (SPR-838, Millar Instruments, Houston, TX, USA) that was inserted into the LV cavity through the right carotid artery. The pressure and volume of the LV were continuously monitored for correct positioning of the catheter. The catheter was coupled to a PowerLab 8/30 A/D converter (AD Instruments; Mountain View, CA, USA) and a personal computer. Parallel conductance correction volumes were determined after injection of 30% hypertonic saline solution (20 μL). Upon completing the hemodynamic measurements, LV volume correction was determined by using heparinized blood that was obtained from each animal. The blood was calibrated by cuvette, according to the method of Parcher and colleagues [18].

### Histopathological Analysis

After the invasive hemodynamic assessment was completed, the rats were euthanized, and their heart and lung tissues were harvested and weighed. The LV from each rat was dissected, and a mid-ventricle slice (~ 3 mm) from each was preserved in 4% paraformaldehyde and embedded in paraffin. Tissue sections (4μm) were stained with Masson’s trichrome and Picrosirius Red to assess fibrosis and collagen deposition, respectively. Histological images were acquired using an optical light microscope with a 2.5× lens (Imager A2 Axio Carl Zeiss, Germany). On average, 12 images were needed to cover the entire LV slice. Images were reconstructed to create a single panoramic slice using PTGui software (version 9.1.3, Rotterdam, The Netherlands).

Fibrosis and collagen deposition were quantified using Image Pro Plus software (version 6.0,Warrendale, PA, USA). Data were expressed as a percentage of total tissue per LV panoramic slice. Fibrosis and collagen deposition were also analyzed for the opposite LV wall (remote area).

### Cross-sectional Area of Cardiomyocytes

To assess cardiac hypertrophy, the cross-sectional area (CSA) of the cardiomyocytes was calculated. Briefly, sections of the LV(4μm) were stained with hematoxylin and eosin (H&E) according to Stefanon and collaborators [19]. Typically, 10 to 15 fields of the remote area were analyzed using a40×objective lens and transmitted light. A total of 70 cells were measured for each animal using Image Pro Plus software (version 6.0, Warrendale, PA, USA) for CSA assessment.

### Protein Quantification by Western Blot

Samples of the LV tissue were snap-frozen in liquid nitrogen and stored at -80°C. Total protein extraction was performed using RIPA buffer (1% (w/w) Nonidet P40, 1% (w/w) sodiumdeoxycate, 0.1% (w/v) SDS, 150mmol/L NaCl, 50mmol/L HEPES pH 7.0, 2mmol/L EDTA pH 8.0, 100mmol/L NaF, 10% glycerol, 1.5mmol/L MgCl_2_, 100mmol/L PMSF in ETOH, 200mmol/L sodium orthovanadate, 1 μg/ml aprotin), and samples were homogenized and centrifuged prior to the determination of protein concentration in supernatants as previously described [20]. Protein extracts (125 μg) were separated using 8% or 10% sodium dodecyl sulfate-polyacrylamide gel electrophoresis (SDS-PAGE) and transferred to nitrocellulose membranes. These membranes were incubated with primary antibodies for IkB-α, pIkB-α, TNFR1, TNFR2, and Pro-COL 1A2 (Santa Cruz Biotechnology Inc., Santa Cruz, CA, USA). Alkaline phosphatase-conjugated secondary antibodies were used (Thermo Scientific, West Palm Beach, FL, USA).Bound antibodies were visualized using a biochemiluminescent detection system according to the manufacturer’s instructions (Invitrogen Carlsbad, CA, USA). All immunoblotting signals were normalized to densitometric data obtained for the corresponding membranes stained with Ponceau, as previously described [21, 22]. Blots were imaged using a charge-coupled device (CCD) camera (Gel Logic Imaging System, Rochester, NY, USA).

### Transcription activity assay of NF-κB-Subunits p50 and p65

Activation of NF-κB p50 and p65 in nuclear extracts prepared from LV tissues was assayed using a commercially available kit and a chemiluminescent detection method according to the manufacturer’s instructions (Thermo Scientific).

### Caspase 3/7 Activity

Caspase 3/7 activity was assayed for each LV protein extract (200 mg) according to the manufacturer’s instructions (Caspase/Glo 3/7, Promega, Fitchburg, WI, USA). Data were recorded using a Glomax 20/20 luminometer (Promega).

### Gene Expression

Infarct samples and remote tissues from the LV were subjected to RNA extraction using Trizol reagent (Ambion, USA). RNA was quantified using 260/280 nm absorbance ratio data. Using a High Capacity cDNA Reverse Transcription kit (Applied Biosystems, Carlsbad, CA, USA), total RNA (1 g) was used for reverse transcription reactions. To detect levels of gene expression, real-time PCR was performed using commercially available Taqman primers for IL-1, IL-6, TNF, NF-κB p50, NF-κB p65, and actin (Applied Biosystems, Carlsbad, CA, USA). Detection of actin was used as an internal control.

### Statistical Analysis

All data are reported as the mean ± standard deviation (SD).The Shapiro-Wilk test for normality was performed. Statistical significance was analyzed by using an unpaired *t*-test or Mann-Whitney test when appropriate. Statistical analyses were performed using GraphPad Prism software (for Mac, version 6, San Diego, CA, USA).

## Results

### Animal Weight, LV Weight, LV/Body Weight Index, and LV Fibrosis

**Fig 1:**
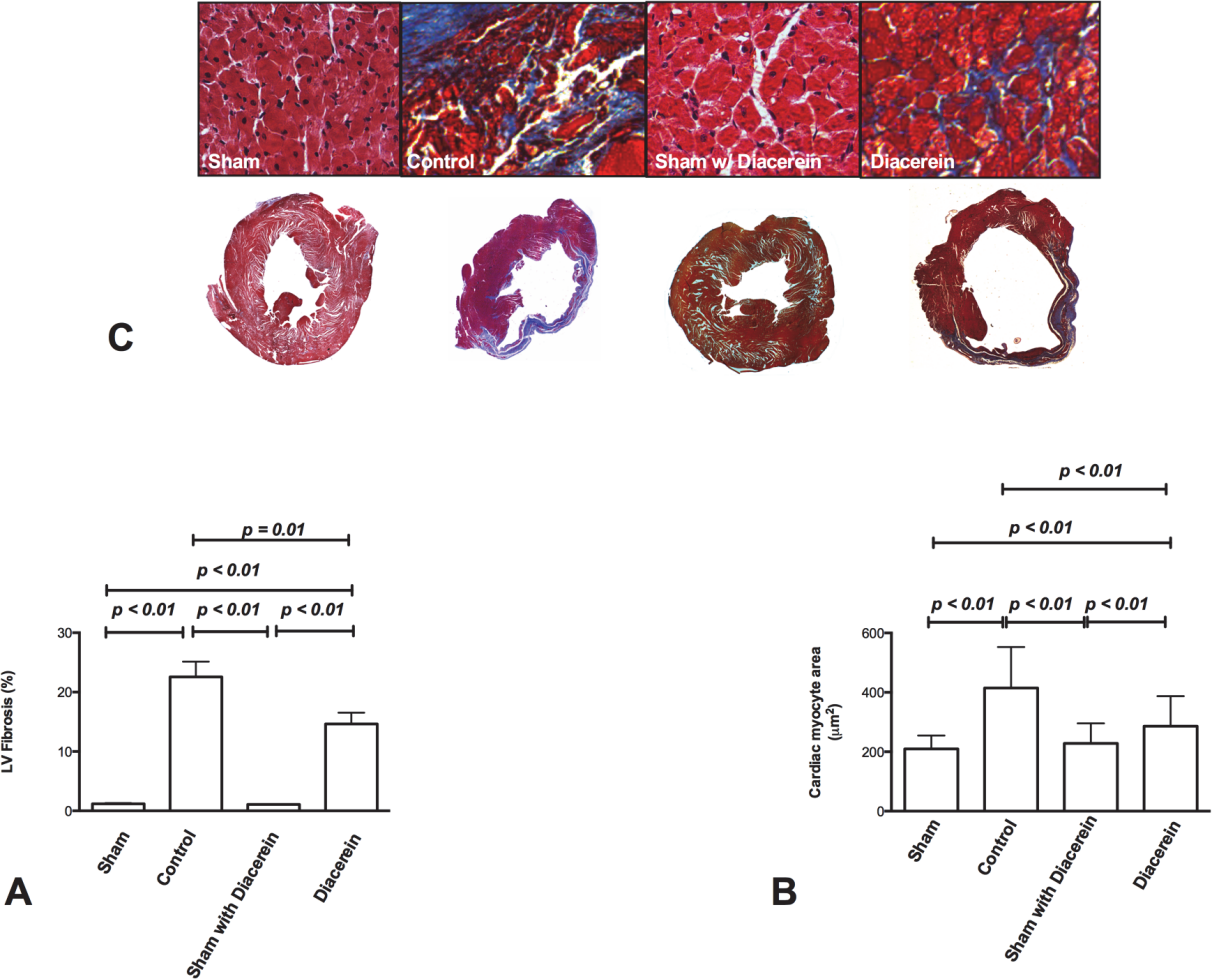
Cardiomyocyte CSA in the remote myocardial region indicated less hypertrophy in the Diacerein group compared to the Control group (A). Fibrosis of the LV was reduced for the Diacerein group (B). Differences between groups were tested by ANOVA test and multiple comparison between groups were tested using Tukey’s test. All values represent the mean ± SE of twelve experiments.

Four weeks after MI, the mean body weight of the Control group was greater than that of the Diacerein group, although both groups exhibited a similar mean weight at the beginning of the study. The Sham group treated with diacerein also exhibited a lower mean body weight compared with the Sham only group. The LV/body weight index values were also higher for the Control and Diacerein groups compared with the Sham and Sham plus diacerein groups 4 weeks post-MI. Using Masson’s trichrome staining, less fibrosis was detected for the Diacerein group compared to the Control group (Fig. 1A) and lower collagen content was observed with Picrosirius Red staining (Table 1). Morphometric analysis further revealed that the Diacerein group had a lower cardiomyocyte CSA compared to the Control group in the remote LV area (Fig. 1B).

**Table 1:** Body weight, lung weight, LV weight, and fibrosis.

Parameter	Sham Group (n = 8)	Control group (n = 16)	Sham Group with Diacerein (n = 10)	Diacerein group (n = 10)	P-value	
Initial BW (±g)	132.23 ± 25.10	144.67 ± 30.10	130.83 ± 7.90	128.73 ± 23.89		.11
BW after 4 wk (±g)	340.30 ± 24.31	282.78 ± 24.48*	272.59 ± 15.25*	257.40 ± 18.38 * ^,^ ^#^		<.01
LV (±g)	0.60 ± 0.04	0.68 ± 0.10	0.59 ± 0.09	0.64 ± 0.05		.05
LV/BW × 1000	1.79 ± 0.14	2.38 ± 0.20 *	1.88 ± 0.15^#^	2.51 ± 0.24 * ^,^ ^$^		<.01
LW/BW × 1000	3.93 ± 0.84	6.91 ± 2.28*	4.1 ± 0.33^#^	6.80 ± 1.93* ^,^ ^$^		<.01
LV fibrosis, %	1.79 ± 0.36	22.57 ± 8.94 * ^,^ ^$^	1.10 ± 0.35^#^	14.65 ± 7.27 * ^,^ ^#^ ^,^ ^$^		<.01
LV collagen, %	0.81 ± 0.32	12.35 ± 6.38*	0.87 ± 0.29^#^	9.08 ± 6.77* ^,^ ^$^		<.01
Lateral wall fibrosis, %	0.68 ± 0.48	3.99 ± 2.88*	0.60 ± 0.46^#^	3.61 ± 2.48* ^,^ ^$^		<.01

BW indicates body weight; LW, lung weight. Data are expressed as mean ± SD. Differences between groups were tested by ANOVA test.

* P <. 05 vs. Sham group.

# P <. 05 vs. Control group.

$ P <. 05 vs. Sham group with Diacerein using Tukey’s test for multiple comparisons.

### Diacerein Promotes Better Hemodynamics 4 Weeks after MI

When lower end-diastolic and end-systolic volumes were monitored, improved LV remodeling was observed for the Diacerein group compared to the Control group. The ejection fraction was also higher for the Diacerein group compared to the Control group. The Control group showed higher dP/dt max and heart rate values compared to the Diacerein group. However, when dP/dt max was normalized to the end-diastolic volume (EDV), the latter of which is a more reliable contractility index [23, 24], higher contractility index values were observed compared to the Control group. In contrast, the Sham group treated with diacerein exhibited lower contractility index values compared with the Sham group, and higher values compared with the Control group. These data are summarized in Table 2.

**Table 2:** Hemodynamic data.

Parameter	Sham Group (n = 8)	Control group (n = 16)	Sham Group with Diacerein(n = 10)	Diacerein group (n = 10)	P-value
TAU, ms	10.56 ± 1.73	16.60 ± 6.36*	11.36 ± 1.83^#^	17.43 ± 4.86*	<.01
dP/dt max, mmHg/s	7835 ± 1172	6288 ± 1678	4408 ± 799* ^,^ ^#^	4714 ± 2072* ^,^ ^#^	<.01
dP/dt min, mmHg/s	-8360 ± 1606	-4550 ± 1288*	-3450 ± 635*	-3698 ± 1653*	<.01
dP/dt max/EDV, mmHg/sμL^-1^	170.20 ± 25.47	50.17 ± 13.39*	93.42 ± 16.93* ^,^ ^#^	75.08 ± 33.00* ^.^ ^#^	<.01
HR, bpm	277 ± 53	311 ± 86	244 ± 37^#^	247 ± 20^#^	.02
Stroke volume, μL	16.03 ± 4.28	41.51 ±26.61*	16.12 ± 4.02^#^	42.28 ± 21.22* ^,^ ^$^	<.01
EDV, μL	46.02 ± 26.69	125.3 ± 69.83*	47.18 ± 25.82^#^	62.78 ± 52.64^#^	<.01
ESV, μL	35.55 ± 26.66	110.6 ± 66.52*	40.22 ± 25.50^#^	46.83 ± 66.03^#^	<.01
EF, %	51.07 ± 33.71	23.21 ± 17.93*	46.85 ± 28.69^#^	50.86 ± 20.39^#^	.03

TAU indicates time constant of isovolumic relaxation; HR, heart rate; bpm, beats per minute; EDV: end-diastolic LV volume; ESV: end-systolic LV volume; EF: ejection fraction of LV.Data are expressed as mean ± SD. Differences between groups were tested by ANOVA test.

* P <. 05 vs. Sham group.

^#^ P <. 05 vs. Control group.

^$^ P <. 05 vs. Sham group with Diacerein using Tukey’s test for multiple comparisons.

### Diacerein Inhibits IκBα and TNF Receptor 1, Modulates NF-κB p65 Transcription, and Promotes Lower Levels of Caspase 3/7Activity

**Fig 2:**
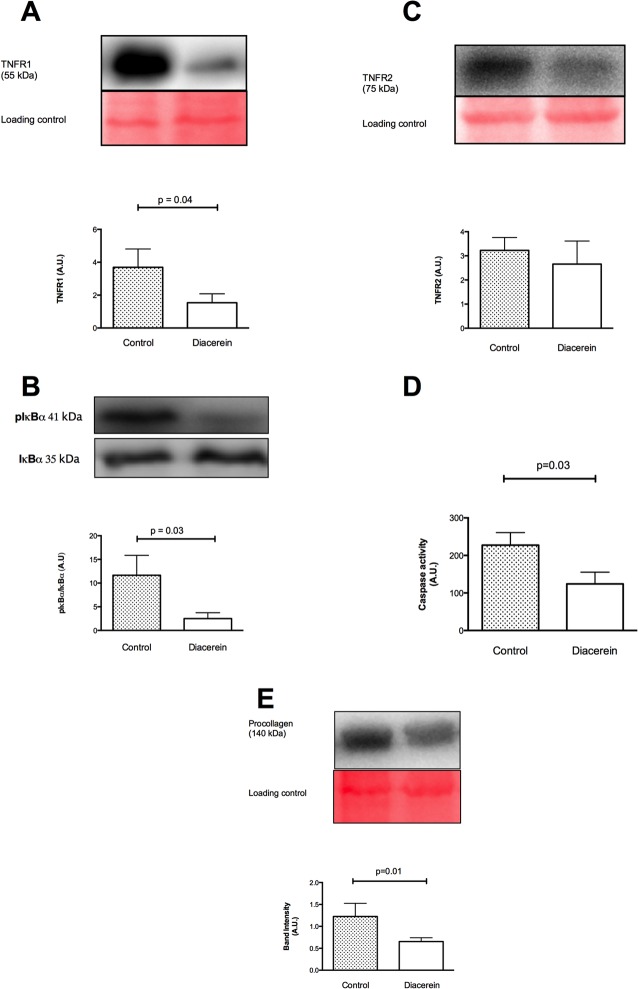
Representative Western blot results that show inhibition of TNFR1 (A) and IκBα (B), no effect on TNFR2 (C), lower caspase 3/7 activity (D), and lower procollagen deposition (E) in the Diacerein group. All values represent means ± SE of five independent experiments. A.U.: arbitrary units.

**Fig 3:**
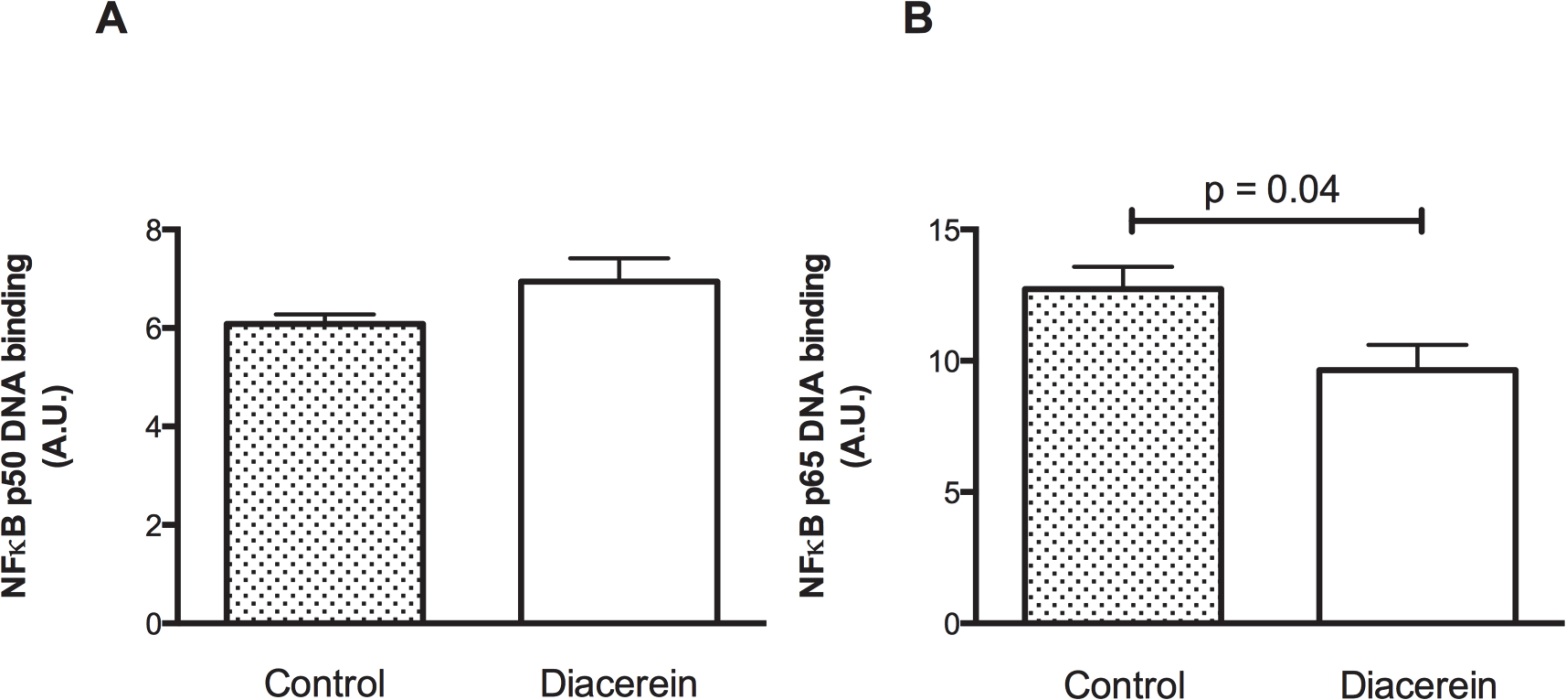
NF-κB transcriptional activity assay of p50 and p65 detected in nuclear extracts collected from tissues in the remote area. All values represent the mean ± SE of five independent experiments. A.U.: arbitrary units.

Lower levels of TNF receptor 1 (TNFR1) expression (Fig. 2A) and IκBα activation (Fig. 2B) were detected for the Diacerein group compared to the Control group 4 weeks after MI. The latter result corresponded to lower levels of NF-κB p65 transcription for the Diacerein group (Fig. 3B). The levels of caspase 3/7 activity (Fig. 2D) and procollagen 1 and 2 deposition (Fig. 2E) were also lower for the Diacerein group compared to the Control group. In contrast, expression levels of NF-κBp50 were similar between the two groups (Fig. 3A).

### Diacerein Promotes Lower *TNF* Gene Expression in Infarcted and Remote Areas of the LV

**Fig 4:**
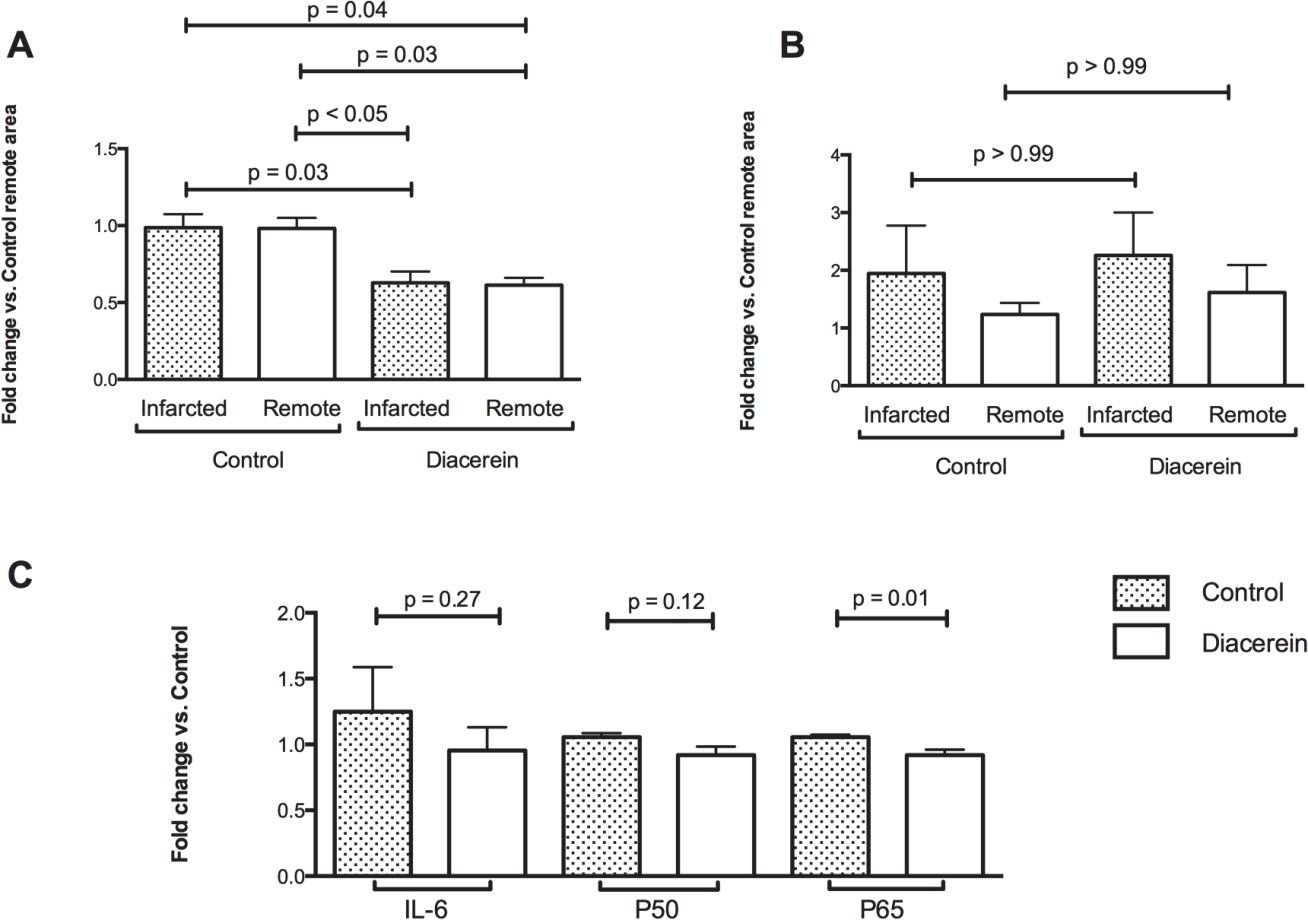
Gene expression detected for TNF (A) and IL-1 (B) in remote and infarcted areas. Gene expression detected for IL-6, NF-κB p50, and NF-κB p65 (C) in the remote areas of both groups. All values represent the mean ± SE of three independent experiments.

After administration of diacerein for 4 weeks, levels of *TNF* gene expression were lower in both remote and infarcted areas of the LV (Fig. 4A). In contrast, levels of *IL-1* gene expression did not significantly differ between the two groups, although lower levels of *IL-1*expression were detected for the Diacerein group (Fig. 4B). In the remote ventricle regions, lower levels of NF-κB p65 gene expression were detected in the Diacerein group compared to the Control group. The NF-κB p50 and IL-6 gene expression were not significantly different in the Diacerein group compared to the Control group (Fig. 4C).

## Discussion

This study demonstrates the beneficial effects of an anti-inflammatory drug, diacerein, on LV remodeling in a rat experimental model of MI. Lower end-systolic and end-diastolic LV volumes, higher LV ejection fractions, less hypertrophy of cardiomyocytes in remote areas, and lower heart rates were detected in rats 4 weeks after an MI event and treatment with diacerein.

Lower dP/dt max values were observed in the treatment group compared to the control and sham groups, and these data suggest that diacerein mediates a detrimental effect on myocardial contractility. However, there were no differences observed after the dP/dt max values were normalized to the EDVs. Lower dP/dt max values were also observed following normalization to EDV values for the Sham group treated with diacerein compared with the Sham group. The latter data may indicate that greater modulation of the parasympathetic system occurred [25]. However, further studies are needed to examine this possibility.

The linearity between dP/dtmax and EDV reported in the literature suggest this index might be a more reliable to assess the contractility state in vivo [23, 26]. Of note, most of the studies that have used dP/dt max as a contractility index had similar heart rate between the groups, which makes the Treppe effect less likely to occur [27, 28]. Ishikawa and colleagues have demonstrated that the ejection fraction is more sensitive at detecting systolic dysfunction over the dP/dt max in an elegant model of myocardial infarction in swine. They suggest the ejection fraction is more suited for inter animal comparisons and dP/dt max is more useful tool to assess contractility within the same animal [24]. Therefore, the present findings demonstrate the diaceiren interfere positively on the LV remodeling after MI, but it was unable to provide any beneficial effect on myocardial contractility state compared to the control group.

These beneficial effects of LV remodeling after MI may have been due to inhibition of TNFR1, which can lead to lower levels of IκBα activation and reduced transcription of NF-κB p65. No significant changes in TNF receptor 2 (TNFR2) were observed during the same experimental period. LV remodeling was also associated with lower levels of caspase 3/7 activity, less fibrosis, less collagen, and reduced procollagen deposition. To our knowledge, the effects of diacerein on LV remodeling after MI have not previously been investigated. The present findings suggest that modulation of the inflammatory response is a key factor in the healing process, in accordance with several reports in the literature [3, 4, 11, 29], supporting the potential clinical utility of a drug with anti-inflammatory effects after MI.

Remodeling of the LV after MI is a complex process that includes several pathways that mediate biochemical, molecular, and morphological alterations in remote and infarcted myocardial regions [19]. The role of innate immunity in this process is not completely understood. However, it appears that TNF mediates bimodal effects, ranging from tissue injury to tissue repair, during the acute and chronic stages after MI[3, 30]. In the present study, selective inhibition of TNFR1 was identified as a potential mechanism by which inhibition of IκBα could regulate the NF-κB p65/p50 heterodimer [31]. This hypothesis is supported by the results of other studies involving TNFR1 [30, 32, 33]. For example, Hamid and colleagues subjected TNFR1^-/-^ and TNFR2^-/-^ mice to MI. They observed disparate and opposing effects on LV remodeling, hypertrophy, and NF-κB activation, with TNFR1 exacerbating the effects of MI and TNFR2 improving the response to MI. In the present study, lower levels of TNFR1 expression were observed with diacerein administration, accompanied by lower levels of fibrosis, caspase 3/7 activity, and NF-κBp65. These results are consistent with those of the TNFR1^-/-^ model [30].

Onai and colleagues previously demonstrated that non-selective blockage of NF-κB improves LV remodeling after MI in their studies of IMD-0354 and IKK-β phosphorylation[8]. In the present study, selective blockage of TNFR1 resulted in an attenuation of NF-κB p65 transcription, lower levels of caspase 3/7 activity, and improved remodeling of the LV 4 weeks after treatment with diacerein. TNFR1 has previously been shown to activate TNFR1-associated death domain (TRADD), which is involved in mediating apoptosis via recruitment of caspase 8 and cleavage and activation of caspase 3, and also contributes to the activation of NF-κB [34]. Onai et al. did not evaluate the effects of IMD-0354 on the NF-κB subunits. However, Hamid and colleagues reported that persistent inhibition of NF-κB p65, coupled with negligible NF-κB p50 activation/inhibition, resulted in improved survival and cardiac remodeling. Decreased pro-inflammatory, pro-fibrotic, and anti-apoptotic effects were observed in transgenic mice that over expressed a mutation for phosphorylation-resistant IκB-α and were subjected to coronary ligation [31]. The latter results are consistent with those of the present study, although lower levels of TNF expression were also accompanied by an absence of changes in *IL-1* and *IL-6* gene expression in the present study.

Several reports have shown that NF-κB plays an important role in cardiac remodeling after MI in animal models and humans [19, 35, 36]. Furthermore, it has been demonstrated that the NF-κB subunits p50 and p65 translocate to the nucleus up to 24 hours after infarction, yet only the p65 subunit is consistently stimulated in animals that experience poor LV remodeling and heart failure [35]. The relationship between innate immunity and LV remodeling after MI remains unclear, although it appears that chronic activation of NF-κB is detrimental to remodeling of the LV [3].Three genetic programs have been identified as being controlled by the NF-κB pathway, with the timing and type of NF-κB activation being key events [37]. These programs include hypertrophy, acute cardiomyocyte protection from ischemia/reperfusion, and chronic cardiomyocyte injury due to a prolonged inflammatory response [30, 31, 37]. In all three programs, chronic activation of NF-κB appears to represent a maladaptive process that perpetuates the inflammatory process and compromises repair of LV function due to the presence of increased fibrosis, reduced myocardial contractility, increased hypertrophy in remote areas, and deterioration of diastolic function [37].

Recently, Gao and colleagues showed a decrease in IκBα phosphorylation in RAW 264.7 cells pretreated with diacerein and stimulated with lipopolysaccharide (LPS)[38]. The decrease in IκBα phosphorylation was also accompanied by a marked decrease in nuclear levels of NF-κB p65 compared to the cells that were not pretreated with diacerein [38]. These observations in an *in vitro* model are very similar to those described here in an animal model.

In the present study, diacerein partially blocked the inflammatory process after MI by inhibiting the NF-κB p65 subunit. This effect was associated with improved remodeling of the LV over a period of 4 weeks post-MI. However, different doses of diacerein were not evaluated to investigate a potential dose-dependent effect. The dose administered was used in a previous study [17].

There were limitations associated with the present study. First, it is possible that the observed decrease in contractility in the diacerein group was due to greater modulation of the parasympathetic system. The current data do not address this and further studies are needed. Second, a clear beneficial effect of diacerin on left ventricle remodeling was observed in the present study in the Western blot assays performed. Lower levels of p-IκBα and nuclear NF-κB p65 were detected in the diacerein group, and similar findings have been reported by Gao and colleagues [38]. Third, all of the Western blots were performed using total tissue extracts instead of cytosolic fraction extracts, and this may explain the similar observations made regarding IκBα expression levels.

## Conclusions and Clinical Implications

In the presence of diacerein, a certain degree of inflammatory blockage, although not complete, was observed in the rat MI model investigated. This blockade may have contributed to the beneficial effects that were associated with LV remodeling. Given that there are several factors involved in cardiac remodeling, it is encouraging to find that inhibition of NF-κB is capable of influencing many aspects of this process. Thus, administration of an NF-κB inhibitor may represent a reasonable therapeutic approach for the treatment of MI.
